# Translational Approaches Targeting Ceramide Generation From Sphingomyelin in T Cells to Modulate Immunity in Humans

**DOI:** 10.3389/fimmu.2019.02363

**Published:** 2019-10-11

**Authors:** Claudia Hollmann, Teresa Wiese, Fabio Dennstädt, Julian Fink, Jürgen Schneider-Schaulies, Niklas Beyersdorf

**Affiliations:** ^1^Institute for Virology and Immunobiology, University of Würzburg, Würzburg, Germany; ^2^Institute of Organic Chemistry, University of Würzburg, Würzburg, Germany

**Keywords:** sphingolipids, CD4^+^ T cells, regulatory T cells (Treg), CD8^+^ T cells, anti-depressant drug

## Abstract

In T cells, as in all other cells of the body, sphingolipids form important structural components of membranes. Due to metabolic modifications, sphingolipids additionally play an active part in the signaling of cell surface receptors of T cells like the T cell receptor or the co-stimulatory molecule CD28. Moreover, the sphingolipid composition of their membranes crucially affects the integrity and function of subcellular compartments such as the lysosome. Previously, studying sphingolipid metabolism has been severely hampered by the limited number of analytical methods/model systems available. Besides well-established high resolution mass spectrometry new tools are now available like novel minimally modified sphingolipid subspecies for click chemistry as well as recently generated mouse mutants with deficiencies/overexpression of sphingolipid-modifying enzymes. Making use of these tools we and others discovered that the sphingolipid sphingomyelin is metabolized to ceramide to different degrees in distinct T cell subpopulations of mice and humans. This knowledge has already been translated into novel immunomodulatory approaches in mice and will in the future hopefully also be applicable to humans. In this paper we are, thus, summarizing the most recent findings on the impact of sphingolipid metabolism on T cell activation, differentiation, and effector functions. Moreover, we are discussing the therapeutic concepts arising from these insights and drugs or drug candidates which are already in clinical use or could be developed for clinical use in patients with diseases as distant as major depression and chronic viral infection.

## Introduction

Subsets of T cells are major contributors to adaptive immunity. In particular, CD4^+^ T helper and CD8^+^ T cells either crucially orchestrate adaptive immune response or are direct mediators of e.g., anti-viral immunity, respectively. In order to be able to fulfill these tasks T cell precursors have to run through a stringent process of positive and negative selection within the thymus [reviewed in (1)]. However, it has been clear for decades that the process of negative selection does not completely eliminate maturing autoreactive T cells. This means that also in healthy human individuals autoreactive T cells can be detected (2–5) which, however, only cause autoimmune diseases like multiple sclerosis in very few people. One reason for this is that the thymus also generates so-called regulatory CD4^+^ T cells—a process coined the “third function” of the thymus (6).

These regulatory T cells (Treg) develop and are maintained under the control of the transcription factor Foxp3 (7–9). Expression of Foxp3 endows maturing T cells with an increased robustness toward negative selection (10). Therefore, Foxp3^+^ CD4^+^ Treg leaving the thymus display a high degree of autoreactivity (11, 12). By employing a wide array of molecular mechansims Treg prevent autoreactive and autoaggressive conventional CD4^+^ T helper (CD4^+^ Tconv) and CD8^+^ T cells from attacking healthy tissue which would otherwise lead to autoimmune disease [reviewed in (13)].

In conditions under which a protective adaptive immune response is crucial for the host to survive, mechanisms need to be in place which neutralize Treg-mediated immunosuppression [reviewed in (13)]. A key mechanism here is the recognition of pathogen-associated molecular patterns (PAMPs) via pattern recognition receptors (PRR) like Toll-like receptors expressed by cells of innate immunity like dendritic cells (DC) [reviewed in (14)]. This leads to an upregulation of costimulatory molecules like CD80 and CD86 on the surface of DC, which will trigger CD28 costimulation of CD4^+^ Tconv. By this they will escape suppression by Treg (15–17). As these signals are spatially and timely restricted, i.e., only present in lymph nodes draining an infection site, Treg-mediated immunosuppression will only be neutralized there, whereas in other tissues Treg will continue to be able to mediate protection from autoimmunity.

Unfortunately, “overshooting” or unwanted adaptive immune responses are not always prevented successfully. This will then lead to different forms of T [reviewed in (18–20)] or B cell-mediated autoimmunity [reviewed in (21, 22)].

Apart from mediating protective immunity and inducing autoimmune diseases causing a substantial amount of morbidity and mortality, T cells have been recognized to play an important role in maintaining or restoring tissue homeostasis after muscle damage (23), myocardial infarction (24–26) or stroke (27).

**Figure 1:**
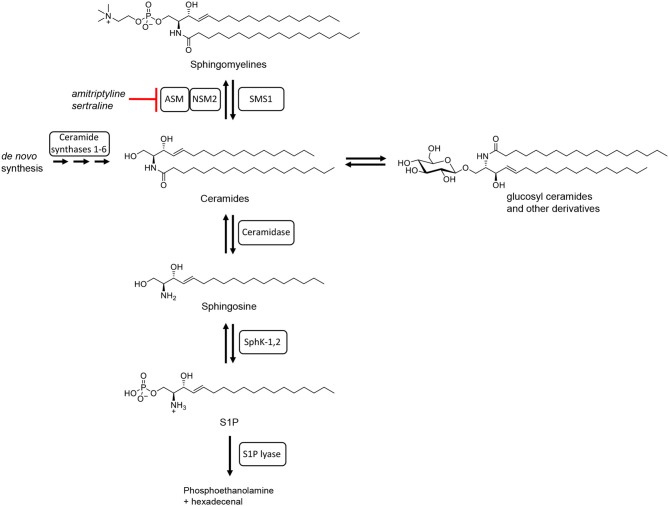
Schematic summary of sphingolipid metabolism. Adapted from Bartke and Hannun (30) with chemical structures of the sphingolipids involved (all with C18 side chains).

To fulfill all these different tasks, T cells in general mainly rely on signals which they receive through cell surface receptors. As such these receptors are, of course, in close contact with lipids forming the cell membrane. About 30% of phospholipids in the plasma membrane belong to so-called sphingolipids [reviewed in (28)]. The importance of sphingolipids for T cell function stems from the fact that they are not inert molecular species, but that they are subject to metabolization [reviewed in (29)] and are altered depending on the differentiation state and function of the cells. This means e.g., that the most complex sphingolipid sphingomyelin (consisting of a number of species with different fatty acid chain lengths) can be reversibly cleaved into ceramides and phosphocholine (Figure 1). Ceramide molecules have the propensity to self-aggregate, thus, forming so-called ceramide-rich platforms [reviewed in (31)]. Ceramides may, however, also be further metabolized into sphingosine and fatty acids. Sphingosine may then be phosphorylated to sphingosine-1-phosphate which has very wide-ranging biological activities mediated by a set of five different cell surface receptors, but also by direct interaction with signaling molecules inside cells [reviewed in (32)]. Finally, sphingosine may also be cleaved into phosphoethanolamine and hexadecenal marking the only non-reversible step in sphingolipid metabolism (Figure 1).

The different steps in the meta-(cata-)bolism of sphingomyelin and its breakdown products are catalyzed by a whole array of different enzymes. Localization of these enzymes in different cellular compartments and the modulation of their enzymatic activity upon T cell activation mean that their biology is very complex. One key mediator of sphingomyelin breakdown is the acid sphingomyelinase (mouse: Asm; human: ASM). In resting T cells the Asm is localized in the inner leaflet of the lysosomal membrane where the presence of Zn^2+^ ions and the acidic pH ensure optimal enzymatic activity [reviewed in (29)]. Upon activation of certain cell surface receptors, including CD28 (33) and CD95 (34) via monoclonal antibodies, Asm activity in lysosomes is increased and lysosomes fuse with the cell membrane, thus exposing the Asm on the cell surface where it might still be able to catalyze sphingomyelin cleavage [reviewed in (29)]. Another important sphingomyelinase whose role in T cell biology is not yet fully understood is the neutral sphingomyelinase 2 (mouse: Nsm2; human: NSM2). In contrast to the Asm, the Nsm2 localizes to the inner leaflet of the plasma membrane and the cytoplasmic side of the Golgi membrane, where it can get activated without being translocated to another cellular compartment (35–38). Stimuli activating the Nsm2 include isolated TCR or TCR and CD28 costimulation via monoclonal antibodies (39).

Apart from meta- and catabolism sphingolipid concentrations in cellular membranes are, of course, also regulated by *de novo* sphingolipid generation. The “hub” of sphingolipid *de novo* synthesis is ceramide [reviewed in (30)] with six different ceramide synthases catalyzing the generation of the various ceramide species in the endoplasmic reticulum [reviewed in (30, 40)].

## CD8^+^ T Cells

Having passed thymic selection mature, MHC class I-restricted, CD8^+^ T cells leave the thymus and migrate to secondary lymphoid organs, i.e., predominantly lymph nodes and spleen. To ensure tight immunological control of the whole body, naïve (CD8^+^) T cells constantly recirculate through the lymphatic/blood system—a process crucially regulated by high concentrations of sphingosine-1-phosphate in efferent lymph and blood [reviewed in (32)].

After encounter of antigen and appropriate costimulation, CD8^+^ T cells differentiate into cytotoxic T lymphocytes (CTL) expressing lytic granules [reviewed in (41)]. Upon recognition of foreign peptides on MHC class I molecules by the CTL's TCR the CTL will release the content of the lytic granules toward the target cell, i.e., into the synaptic cleft between both cells. Lytic granules contain granzymes and perforin which generates pores in the target cell's membrane through which granzymes can enter the cytoplasm and induce apoptosis by activating caspases [reviewed in (42)]. Upon fusion of the lytic granules with the cell membrane not only proteins like LAMP-1 which is expressed on the inner membrane leaflet of lytic vessels and protects CTL and Natural Killer cells from degranulation-associated damage (43), but also the Asm will be exposed on the cell surface [reviewed in (29, 44)]. Due to the size of the lytic granules sufficient extrusion of their content requires changes to the biophysical properties of their membranes (45). These changes are mediated by the Asm generating ceramide at the inner membrane leaflet of the vesicles. Vesicles containing the chemokine RANTES which also exist in CD8^+^ effector T cells are about 10-fold smaller than lytic vesicles. Therefore, chemokines are efficiently released from these vesicles even without changes to the biophysical properties of their membranes (45). Reduced release of lytic content from vesicles was associated with reduced killing by CTL from Asm-deficient vs. wild-type mice (45). These data were confirmed by pharmacologically inhibiting Asm activity with imipramine in CTL (45).

Can these insights be used and translated to humans? A direct consequence of the aforementioned observations in mice is that inhibition of Asm activity with clinically approved antidepressants like amitriptyline, imipramine, or sertraline could reduce unwanted CTL activity. This would, of course, lead to unspecific partial immunosuppression. It might, thus, be envisaged as a form of comedication e.g., in patients suffering from pulmonal immunopathology due to overshooting CTL activity against e.g., Influenza A virus-infected alveolar epithelial cells [reviewed in (46)]. In addition to reducing CTL activity, inhibition of the ASM in humans may also directly stabilize pulmonal function as it has been observed in animal models (47–49). In fact it is this latter indication for which ASM inhibitors are currently investigated in children with cystic fibrosis suffering from bacterial infections of the lung (ClinicalTrials.gov Identifier: NCT00515229).

A caveat in the outlined scenario is that, so far, the role of the Asm in lytic granule release from CTL has only been studied in mice. Data for human CD8^+^ CTL are still lacking. As it is very likely that the ASM is also expressed by human CD8^+^ T cells it seems plausible that also in humans ASM activity enhances release of cytotoxic content from CTL vesicles.

Apart from the ASM, the NSM2 also constitutes a therapeutically interesting target to modulate CTL function in humans. This is the case as stimulation of the T cell receptor complex (with monoclonal antibodies) is sufficient to increase NSM2 activity (39) and for target cell recognition the CTL only needs to receive an activating signal through the TCR complex. Therefore, inhibition of the NSM2 may also be suitable to reduce unwanted CTL activity.

## Conventional CD4^+^ T Cells

Compared to CD8^+^ T cells and CTL, the body of literature on the impact of sphingolipid metabolism on the function of conventional, i.e., Foxp3^−^, non-regulatory T cells (CD4^+^ Tconv) is much bigger (50–52). In particular, researchers have focused in recent years on the modulation of effector cell differentiation and cytokine secretion by human CD4^+^ T cells by the ASM (52, 53).

An early report comparing cells from Asm-deficient and wildtype mice indicated that secretion of Interleukin-2 (IL-2) by Concanavalin A-stimulated splenocytes among which CD4^+^ Tconv are probably the main, followed by CD8^+^ T cells, source of IL-2 [reviewed in (54)], was higher in wild-type than in mutant mice (55). More recently, experiments using human CD4^+^ T cells have revealed a positive role for the ASM in promoting Th17 cell differentiation and IL-17 secretion (52). Here, pharmacological inhibition of the ASM was used to study the impact of the enzyme on CD4^+^ T cell differentiation and cytokine secretion.

Apart from cytokine secretion, migration of effector/memory CD4^+^ Tconv also crucially contributes to their function *in vivo*. Studying mouse CD4^+^ T cells *in vitro* and *in vivo* as well as human CD4^+^ T cells *in vitro*, it has been recently shown that migration and adhesion to activated endothelial cells requires NSM2 activity (56). Moreover, migration of T cells toward SDF-1α, a chemokine recognized by CXCR4, also depends on NSM2 activity (56). Therefore, two crucial steps in CD4^+^ effector cell function, i.e., extravasation at sites of endothelial inflammation, and migration along chemokine gradients necessitates NSM2 activity. For extravasation integrin leukocyte function-associated antigen (LFA)-1 on T cells needs to bind to intercellular adhesion molecule (ICAM)-1 on endothelial cells. Therefore, reduced LFA-1 clustering in the absence of NSM2 activity (56) should impact all T cell subsets. Similarly, the broad expression of CXCR4 by (CD4^+^) T cells [reviewed in (57)] also means that targeting NSM2 in T cells affects early as well as advanced stages of T cell differentiation. Apart from its impact on T cell migration NSM2 activity also supports early signaling events in Jurkat and primary human CD4^+^ T cells (58). In the absence of NSM2 activity, T cell receptor signaling is initiated as in wild-type T cells, but signaling is not sustained due to deficient protein kinase Cς activation. Together, this means that the NSM2 might qualify as a novel therapeutic target for suppressing unwanted immune responses. Currently, there is, however, no data concerning the immunomodulatory activities of NSM2 inhibitors in humans *in vivo*.

Another critical aspect of T cell biology is the tight homeostatic control of the compartment size through induction of different forms of cell death [reviewed in (59)]. Most notably, activation of naïve (CD4^+^) T cells is followed by massive expansion of reactive clones. After resolution of inflammation the effector T cell pool again collapses with only few memory T cells surviving long-term [reviewed in (60, 61)]. The collapse of the acute immune response is due to different mechanisms of cell death with apoptosis induction by Fas (CD95)-Fas ligand being the best studied pathway, but other forms of cell death like necroptosis are increasingly recognized to also play a role here [reviewed in (59, 62)]. Ligation of Fas on activated T cells stimulates Asm activity leading to ceramide production and, as a consequence, to the induction of cell death (63–65). Therefore, and even more generally, ceramide production has been linked to induction of cell death [reviewed in (29)]. Seemingly in contrast to this notion we observed that pharmacological inhibitors of the Asm also induced cell death in Tconv of mice (66) and at slightly higher concentrations also in human CD4^+^ Tconv (Dennstaedt, Schneider-Schaulies, Beyersdorf, unpublished). The availability of Asm-deficient mice allowed to confirm that cell death induced by amitriptyline or desipramine was due to their impact on the Asm and not the acid ceramidase which they also inhibit (66, 67). This has, as discussed in the following paragraph, an impact on the balance of CD4^+^ Tconv and Treg.

In patients treated with antidepressants inhibiting ASM activity like amitriptyline or sertraline (68) no (CD4^+^) lymphopenia has been reported. This might be due to the relatively low concentrations of ASM-inhibiting antidepressants in peripheral blood of humans (about 1 μM) (69). In secondary lymphoid organs it is, however, assumed that up to 10-fold higher concentrations are reached (69). As ASM inhibitors are sufficient to kill human CD4^+^ Tconv *in vitro* this indicates that ASM inhibitors might also induce cell death in human CD4^+^ Tconv in secondary lymphoid organs *in vivo*. For mice we had observed that the negative effects on CD4^+^ Tconv cell numbers in spleen and lymph nodes after Asm inhibition *in vivo* were less pronounced than after *in vitro* treatment of mouse T cells (66). Similar to patients, serum concentrations of amitriptyline in these mice were also in the order of 1 μM (70). This might indicate that there are pro-survival factors present *in vivo* which were lacking in the *in vitro* cell cultures. But despite such putatively beneficial factors, a reduction in CD4^+^ Tconv numbers in spleens and less so in lymph nodes was observed (66), which is best explained by induction of cell death in a fraction of these cells. Therefore, in humans *in vivo* CD4^+^ Tconv depletion might also take place on a small scale. Due to the long-term use of antidepressants by patients and the very low output rate of the thymus in adults (71) it may well be that these patients gradually become lymphopenic over time.

## Treg

Treg differ from CD4^+^ Tconv and CD8^+^ T cells in that, due to their autoreactivity (11, 12), they constantly receive activating signals through their T cell receptor—even in healthy subjects. Apart from the T cell receptor, signaling through CD28 and the high affinity IL-2 receptor are crucial to maintain Treg numbers and function (72–77). For CD28 it has been shown that ligation with monoclonal antibodies strongly increases ASM activity in human T cells (33). In line with their dependence on CD28 signaling for survival, both mouse (66) and human Treg (Dennstaedt, Schneider-Schaulies, Beyersdorf, unpublished) show constitutively higher ASM activity than CD4^+^ Tconv and for mouse Treg it has been shown that they also contain increased amounts of ceramide compared to CD4^+^ Tconv (66, 78).

Apart from the Asm, underexpression of the sphingomyelin synthase 1 (Sms1) in Treg vs. CD4^+^ Tconv contributes to the increased ceramide content of Treg vs. CD4^+^ Tconv (78). The increase in ceramide induced by this lack of Sms1 maintains suppression of the Akt/mTOR pathway in Treg through the phosphatase PP2A. Suppression of Akt/mTOR signaling is crucial for Treg to be able to inhibit CD4^+^ Tconv (78).

Currently, it is unclear whether or how much the ceramide pools regulated by Asm and Sms1 activity overlap. This is of importance as for the activation of the phosphatase PP2A its inhibitor SET needs to bind to ceramide (79). The Asm is expressed in the inner leaflet of the lysosomal membrane and translocates to the outer leaflet of the cell membrane upon T cell activation and fusion of the lsysome with the cell membrane. Therefore, Asm activity generates ceramide in the inner leaflet of the lysosome and the outer leaflet of the cell membrane. However, ceramide spontaneously flips from one membrane leaflet to the other and for other sphingolipid species “filppase”-mediated exchange between membrane leaflets has been described [reviewed in (29)]. As ceramide generated by the Asm may, thus, also accumulate in the cytosolic leaflet of membranes Asm activity might enhance PP2A activity in Treg. Genetic deficiency for the Asm in mice led to an increase in the proportion of Treg among CD4^+^ T cells (66), which would be in line with this hypothesis. However, the suppressive activity of Treg was increased on a per-call basis as read out in surrogate *in vitro* suppression assays (66). This suggest that ceramide generated by the Asm may not be critical for PP2A activity as otherwise suppression by Treg would have been lost (78). More definite conclusions regarding the ceramide pools regulated by the Asm vs. the Sms1 are not possible as the changes in sphingolipid composition in cells of Asm-deficient compared to wildtype mice are very complex. Despite their Asm deficiency, T cells, and other cells, display strongly increased ceramide levels (66, 80, 81). In parallel, the sphingomyelin content of these cells is even further increased (66, 80, 81). This means that the substrate/product ratio for the Asm is reduced in these animals as might be expected due to the Asm deficiency. However, it is currently unclear what exactly drives the changes we observed in these animals with regard to Treg: Whether it is the overall amount of sphingolipids found in these cells or whether it is the sphingolipid composition of membranes.

For Treg a plethora of different molecular mechanisms has been described by which they might inhibit other T cells [reviewed in (13)]. A crucial effector molecule, mediating cell contact-dependent suppression by Treg is the checkpoint molecule CTLA-4 (82–85). Being strongly activated we observed higher CTLA-4 expression in Treg of Asm-deficient vs. wildtype mice (66). CTLA-4 functions as an immune checkpoint by removing costimulatory molecules from the surface of antigen-presenting cells like dendritic cells or B cells (83, 84). This process is called transendocytosis. As the costimulatory receptor CD28 and CTLA-4 share the ligands CD80 and CD86, CTLA-4-mediated transendocytosis leads to a net reduction in T cell costimulation and, thus, immunosuppression. During transendocytosis, the complex of CTLA-4 and bound ligand is internalized and degraded within the lysosome [reviewed in (84)]. In fact, endo-lysosomal vesicles contain the vast majority of CTLA-4 molecules expressed by a T cell under steady-state conditions. Only upon activation CTLA-4 surface expression is increased, primarily within the immunological synapse (86). The low CTLA-4 surface expression is the consequence of shuttling from endo-lysosomal compartments to the cell surface followed by rapid internalization in the absence of ligand binding [reviewed in (84)]. Therefore, the biological activity of CTLA-4 is governed by this complex expression pattern. Using a so-called “capture assay” we monitored CTLA-4 turn-over between the cell membrane and cellular compartments in Treg from wild-type and Asm-deficient mice (66). Here, we observed that Treg from Asm-deficient mice showed a higher turn-over than Treg from wild-type mice.

For human Treg we used pharmacological inhibitors of the ASM to study its impact on CTLA-4 function and turn-over. We observed that inhibition of the ASM in human Treg increased CTLA-4 turn-over as observed in Treg from Asm-deficient mice (Wiese, Schneider-Schaulies, Beyersdorf, unpublished). Therefore, both in mouse and in human Treg, ASM activity is important for the turn-over of CTLA-4.

Although Treg generation is the “third function” of the thymus, Treg may also differentiate from CD4^+^ Tconv in mice under certain conditions (87). The generation of so-called peripherally induced Treg (pTreg) is thought to be of particular importance for immunity in humans [reviewed in (88)]. The identification of two distinct thymic Treg precursors in mice expressing predominantly self-reactive TCRs and TCRs with reactivity to foreign antigen (12), however, challenges this concept and might pinpoint to the thymus as the sole source of *bona fide* Treg also in humans. Together these findings mean that by studying conditions under which pTreg can be generated from CD4^+^ Tconv *in vitro* one analyses the impact of certain factors primarily on the stability of the Treg lineage. Using such *in vitro* systems it was observed that Asm activity has a supportive effect for pTreg generation from Tconv (89).

## Outlook

Up to now antidepressants inhibiting ASM activity are the most widely applied drugs in humans directly impacting on ceramide generation from sphingomyelin (prevalence depression: 5,000/100,000) (90). As these drugs not only induce degradation of the ASM, but also the acid ceramidase (67), more specific direct inhibitors would be clinically desirable. With bisphosphonates such as zoledronate, which are used for the treatment of osteoporosis (prevalence: about 5,000/100,000 in people in their fifties and 25,000/100,000 in octogenerians) (91), safe drugs are available that directly inhibit ASM activity. Currently, it is, however, unclear whether bisphosphonates, including the very potent ASM inhibitor ARC39 (92, 93), will also modulate ASM activity in T cells *in vivo* or whether their high degree of binding to bone surfaces and osteoclasts prohibits sufficient drug levels in secondary lymphoid organs to modulate T cell activity.

The data obtained on the contribution of ASM and NSM2 activity to T cell function in preclinical mouse models and with human T cells *in vitro* all suggest that pharmacologically blocking these enzymes will either directly or, through biasing the CD4^+^ T cell compartment toward Treg, indirectly impair T cell function. Therefore, a potential novel indication for the use of ASM or possibly also NSM2 inhibitors might be autoimmune diseases such as multiple sclerosis (prevalence: about 100/100,000) (94, 95). As discussed, overshooting immunity in the course of e.g., an influenza A virus infection may constitute another potential novel application for ASM inhibitors.

As sphingomyelinase deficiency impairs T cell function boosting sphingomyelinase activity might increase their function which could improve e.g., anti-cancer immunity. Indeed, it has recently been shown that T cell-specific overexpression of the ASM leads to enhanced T cell-mediated immunity against the parasite *Plasmodium yoelii* (96). This suggests that also in humans increasing ASM activity in T cells might enhance T cell-mediated immunity. Therefore, future research should focus on identifying suitable drugs for increasing ASM activity in human T cells.

Growing knowledge on the role of sphingolipid metabolism in T cell biology fuelled by the generation of novel inducible knock-out mouse models as well as novel analytical tools will help to define more potential therapeutic targets. For these, either small molecule or monoclonal antibody-based therapies may allow for specific targeting.

## Data Availability Statement

All datasets generated for this study are included in the manuscript/supplementary files.

## Author Contributions

CH, TW, and FD generated data and edited the paper. JF designed figures. JS-S designed figures and edited the paper. NB wrote the paper.

### Conflict of Interest

The authors declare that the research was conducted in the absence of any commercial or financial relationships that could be construed as a potential conflict of interest.
